# Trends in treatment, incidence and survival of hypopharynx cancer: a 20-year population-based study in the Netherlands

**DOI:** 10.1007/s00405-017-4766-6

**Published:** 2017-10-28

**Authors:** Japke F. Petersen, Adriana J. Timmermans, Boukje A. C. van Dijk, Lucy I. H. Overbeek, Laura A. Smit, Frans J. M. Hilgers, Martijn M. Stuiver, Michiel W. M. van den Brekel

**Affiliations:** 1 Department of Head and Neck Oncology and Surgery, The Netherlands Cancer Institute, Plesmanlaan 121, 1066 CX Amsterdam, The Netherlands; 2 Department of Research, Comprehensive Cancer Organization The Netherlands (IKNL), Utrecht, The Netherlands; 3 Department of Epidemiology, University Medical Center Groningen, University of Groningen, Groningen, The Netherlands; 4 PALGA (The Dutch Nationwide Network and Registry of Histopathology and Cytopathology), Houten, The Netherlands; 5 Department of Pathology, The Netherlands Cancer Institute, Amsterdam, The Netherlands; 6 Institute of Phonetic Sciences-Amsterdam Center of Language and Communication, University of Amsterdam, Amsterdam, The Netherlands; 7 Department of Clinical Epidemiology Biostatistics and Bioinformatics, Academic Medical Center, Amsterdam, The Netherlands; 8 Department of Oral and Maxillofacial Surgery, Academic Medical Center, Amsterdam, The Netherlands

**Keywords:** Hypopharynx cancer, Total laryngectomy, Chemoradiotherapy, Radiotherapy, Survival

## Abstract

Hypopharynx cancer has the worst prognosis of all head and neck squamous cell cancers. Since the 1990s, a treatment shift has appeared from a total laryngectomy towards organ preservation therapies. Large randomized trials evaluating treatment strategies for hypopharynx cancer, however, remain scarce, and frequently this malignancy is evaluated together with larynx cancer. Therefore, our aim was to determine trends in incidence, treatment and survival of hypopharynx cancer. We performed a population-based cohort study including all patients diagnosed with T1–T4 hypopharynx cancer between 1991 and 2010 in the Netherlands. Patients were recorded by the national cancer registry database and verified by a national pathology database. 2999 patients were identified. The incidence increased significantly with 4.1% per year until 1997 and decreased non-significantly afterwards. For women, the incidence increased with 1.7% per year during the entire study period. Total laryngectomy as primary treatment significantly decreased, whereas radiotherapy and chemoradiation increased. The 5-year overall survival significantly increased from 28% in 1991–2000 to 34% in 2001–2010. Overall survival for T3 was equal for total laryngectomy and (chemo)radiotherapy, but for T4-patients the survival was significantly better after primary total laryngectomy (± adjuvant radiotherapy). This large population-based study demonstrates a shift in treatment preference towards organ preservation therapies. The 5-year overall survival increased significantly in the second decade. The assumed equivalence of organ preservation and laryngectomy may require reconsideration for T4 disease.

**Electronic supplementary material:**

The online version of this article (doi:10.1007/s00405-017-4766-6) contains supplementary material, which is available to authorized users.

## Introduction

Despite improvements in radiotherapy (RT) techniques and the advent of chemoradiation (CRT), hypopharynx cancer has the poorest prognosis of all head and neck squamous cell cancers (SCC) [1]. In the US and Europe, it represents approximately 3–14% of all head and neck SCC’s and up to 75% of newly diagnosed patients present in stage III or IV [1–4]. This is in part due to the ‘silent’ anatomical location, resulting in late presentation of symptoms [4]. Furthermore, the hypopharynx has a rich submucosal lymphatic network, which promotes early spread towards lymph nodes [2, 5, 6]. Since the majority of patients are heavy smokers and drinkers, they generally present with multiple co-morbidities [7].

Historically, total laryngectomy (TL) with (partial or total) pharyngectomy used to be the gold standard in hypopharynx cancer treatment. However, since the introduction of CRT in the 1990s there has been a shift towards the use of organ preservation strategies [8, 9]. Randomized controlled trials comparing organ preservation treatment strategies to TL for hypopharynx cancer remain scarce, probably due to the relatively low incidence [1]. Therefore, presently population-based studies give the highest level of evidence to gain insight into the epidemiology and survival. In this study, we investigate the national trends in treatment, incidence and survival for hypopharynx cancer in the period 1991–2010.

## Materials and methods

We conducted a retrospective population-based cohort study based on data retrieved from the databases of the Netherlands Cancer Registry (NCR) and PALGA (the nationwide network and registry of histo- and cytopathology in the Netherlands) [10].

We included all patients diagnosed with T1-T4N0-N3M0 SCC of the hypopharynx in the Netherlands between 1991 and 2010. The following data were retrievable: age at incidence, sex, subsite of tumor according to the International Classification of Disease for Oncology (ICD-0-3) [11], TNM classification [12–16], primary treatment [surgery, RT, chemotherapy (CT)], patient vital status (alive, deceased, lost to follow-up), and follow-up time. The NCR coded type of treatment as RT, CT, surgery or a combination of these. Timing of radiotherapy and chemotherapy was unknown. However, as induction chemotherapy in the Netherlands has never been a standard outside trials, the great majority has been treated with concomitant chemoradiation. By examining the pathological report from the PALGA database, we were able to verify the type of surgery performed and the date. To comply with privacy legislation, both databases were anonymized by a trusted third party; therefore, we were unable to extent our database with additional clinical variables such as co-morbidity, intoxications and exact dose of chemotherapy and radiotherapy, a limitation most population-based studies have.

Our main outcome measures were trends in incidence expressed by European Standardized Rates (ESR), trends in primary treatment and trend in 5-year OS rates. The ESR are rates standardized for the age distribution of a population, which allows for a better comparison between the various European countries and time periods [17].

This study does not fall under the scope of the Medical Research Involving Human Subjects Act, which means it did not have to be approved by an accredited Multicenter Medical Research and Ethics Committee (MREC). The privacy committees of NCR and PALGA foundation approved this study.

### Statistical analysis

We analyzed incidence rates for the period 1989–2013. Using the Joinpoint Regression Program (version 3.5.3. May 2012; Statistical Research and Applications Branch, National Cancer Institute), the estimated annual percentage change (EAPC) of the ESR was calculated using the log-linear model, allowing for a maximum of four joinpoints. To assess changes in treatment and 5-year OS, patients were divided into patients diagnosed in the first decade (1991–2000) or the second decade (2001–2010). We used the Chi square to assess trends in treatment between the two decades. Kaplan–Meier analysis was used to analyze 5-year OS rates. Univariable comparisons were tested using the Log Rank Test. Using the R package cmprsk [18], a competing risk survival analysis was conducted to calculate the cumulative incidence of salvage laryngectomy and death, respectively. Cox Regression analysis was used for multivariable analyses. SPSS^®^ Statistics 20.0 (IBM, Armonk, NY, USA) and R-3.2 [19] were used to perform all the statistical analyses.

## Results

Combining the two national databases resulted in 3016 patients diagnosed with T1-T4N0-N3M0 SCC of the hypopharynx in the Netherlands during the period 1991–2010. We excluded 17 (0.6%) patients because the pathology reports showed that the main location of the tumor was outside the hypopharynx (*n* = 16) or because the pathology report questioned the presence of malignancy (*n* = 1). This left 2999 patients for further analyses. Patient characteristics are shown in Table 1. Most tumors were located in the pyriform sinus (71%), followed by the posterior pharyngeal wall (8%) and the postcricoid area (6%).

**Table 1:** Patient characteristics

	TL (no. %)	RT (no. %)	CRT (no. %)	CT (no. %)	Local sugery (no. %)	No treatment (no. %)	Total (no. %)
**Sex**							
Male	465 (82)	1010 (77)	614 (82)	37 (77)	38 (76)	209 (77)	2373 (79)
Female	102 (18)	301 (23)	138 (18)	11 (23)	12 (24)	62 (23)	626 (21)
**Age in catagories**							
< 50	83 (15)	140 (11)	119 (16)	9 (19)	10 (20)	24 (9)	385 (13)
50–59	175 (31)	365 (28)	309 (41)	20 (42)	17 (34)	62 (23)	948 (32)
60–69	191 (34)	424 (32)	245 (33)	15 (31)	13 (26)	82 (30)	970 (32)
> 70	118 (21)	382 (29)	79 (11)	4 (8)	10 (20)	103 (38)	696 (23)
**TNM classification**							
T1N0	18 (3)	85 (7)	6 (0.8)	0 (0)	17 (34)	10 (4)	136 (5)
T1N+	8 (1)	128 (10)	27 (4)	1 (2)	6 (12)	10 (4)	180 (6)
T2N0	44 (8)	194 (15)	36 (5)	4 (8)	12 (24)	15 (6)	305 (10)
T2N+	48 (9)	273 (21)	145 (19)	5 (10)	4 (8)	22 (8)	497 (17)
T3N0	48 (9)	75 (6)	39 (5)	3 (6)	5 (10)	17 (6)	187 (6)
T3N+	107 (19)	189 (14)	183 (24)	9 (19)	2 (4)	38 (14)	528 (18)
T4N0	101 (18)	111 (9)	69 (9)	6 (13)	3 (6)	47 (17)	337 (11)
T4N+	193 (34)	256 (20)	247 (33)	20 (42)	1 (2)	112 (41)	829 (28)
**Stage grouping**							
Stage I	18 (3)	85 (7)	6 (0.8)	0 (0)	17 (34)	10 (4)	136 (5)
Stage II	44 (8)	194 (15)	36 (5)	4 (8)	12 (24)	15 (6)	305 (10)
Stage III	108 (19)	255 (20)	90 (12)	4 (8)	8 (16)	41 (15)	506 (17)
Stage IV	397 (70)	777 (59)	620 (8)	40 (83)	13 (26)	205 (76)	2052 (68)
**Total**	567	1311	752	48	50	271	2999

*TL* total laryngectomy (with/without (partial) pharyngectomy), *RT* radiotherapy, *CRT* chemoradiotherapy, *CT* chemotherapy

### Trends in incidence

**Fig. 1:**
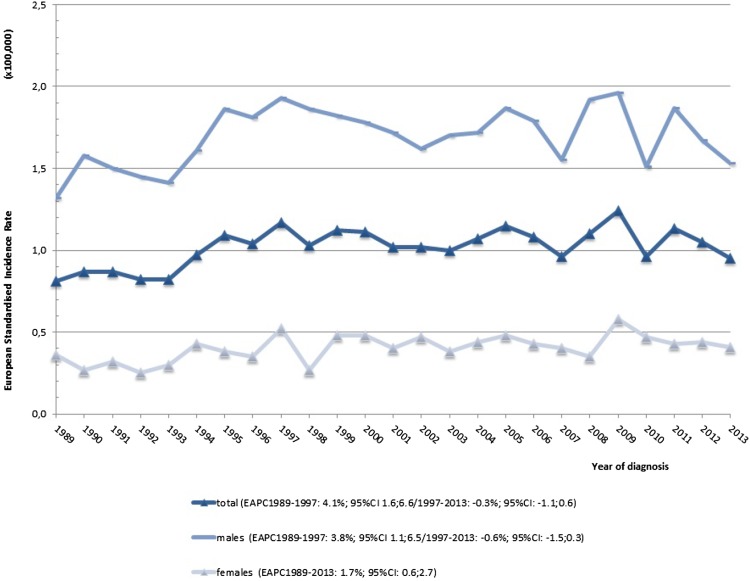
Incidence rate for T1–T4 hypopharynx cancer. The estimated annual percentage change over the standardized incidence and mortality rates (ESR) was calculated with the log-linear model, allowing for a maximum of four joinpoints

Incidence and mortality rates in the Netherlands were analyzed for the period 1989–2013. The total number of patients diagnosed with hypopharynx cancer in the Netherlands increased from 116 in 1989 to 208 in 2013, resulting in an increase in ESR from 0.81 (per 100,000) to 0.95 (per 100,000), respectively. The male incidence declined non-significantly since 1997 but the female incidence rose with 1.7% EAPC since 1989 (*p* < 0.05, Fig. 1).

### Trends in treatment

Overall, the majority of patients were treated with RT (44%), followed by CRT (25%) and primary TL with or without post-operative RT (19%). There was a small and heterogeneous group of patients, who were treated with surgery other than TL (2%). Furthermore, 2% of patients received CT only and 9% of patients were not treated at all.

**Fig. 2:**
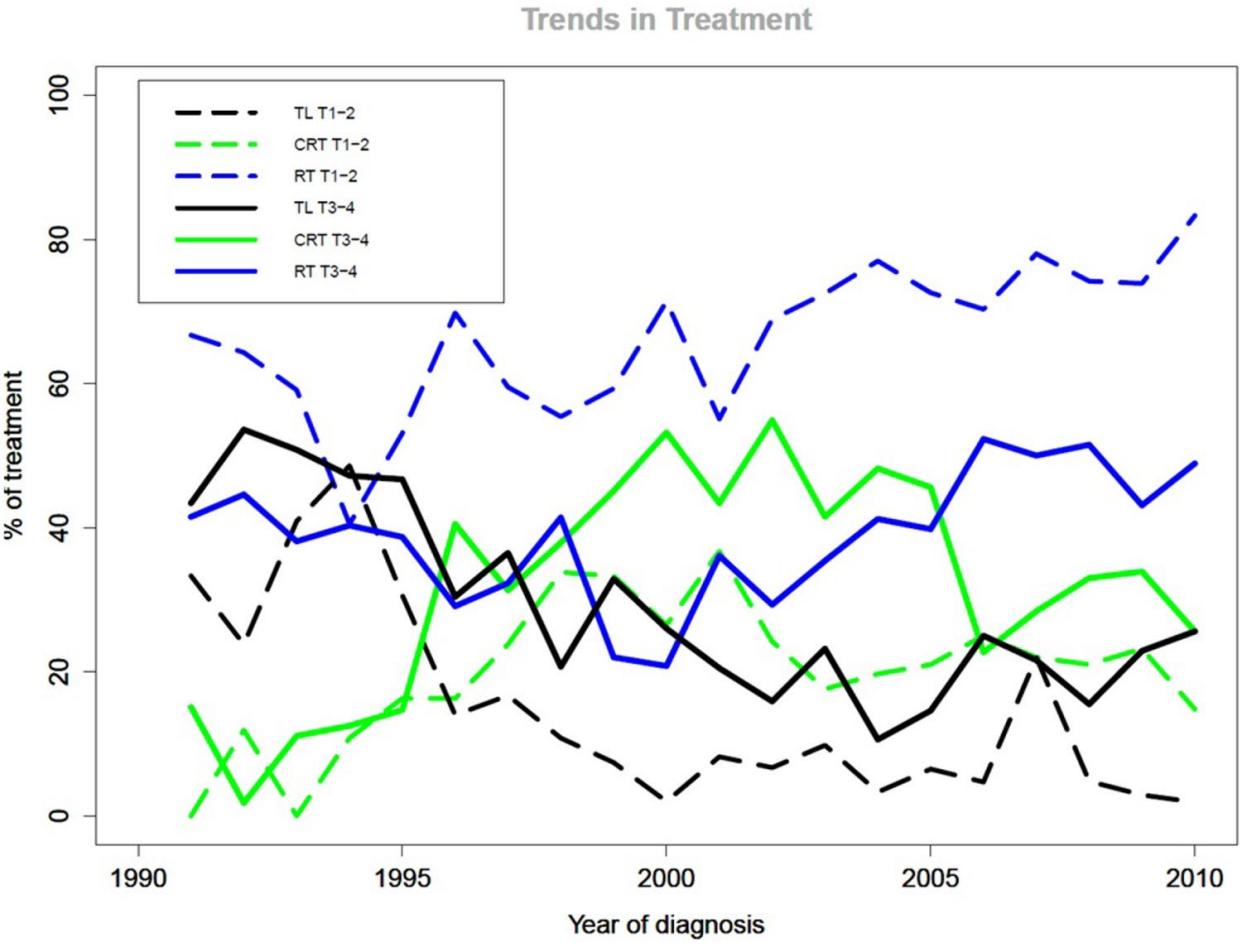
Trends in treatment for T1–T4 hypopharynx cancer. The *X* axis depicts the year of diagnosis; the *Y* axis depicts the primary treatment divided by the total number of patients treated with CRT (green), RT (blue) or TL (black) that year, for T1T2 (dotted lines) and T3T4 (straight lines) in percentages

Figure 2 shows the trends in treatment. During the first decade, 20% of all patients with T1–T2 hypopharynx cancer were treated with TL, which decreased significantly to 4.8% in the second decade (*p* < 0.001). The use of RT in T1–T2 increased significantly from 60% in the first decade to 73% in the second decade (*p* < 0.001). CRT remained more or less stable (20 and 22%, respectively; *p* = 0.27).

For patients diagnosed with T3–T4 hypopharynx cancer, the use of TL decreased significantly from 38% during the first decade to 20% during the second (*p* < 0.001). The largest decline was seen in T3 patients, from 39 to 14%, versus a decline from 38 to 23% for T4 patients (both *p* < 0.001). For T3–T4 patients, RT and CRT both showed a significant increase in the second decade from 34 to 43% for RT (*p* = 0.007) and 28 to 38% for CRT (*p* < 0.001). The number of not-treated patients remained the same, as did the distribution of TNM classification.

### Total laryngectomy

Of the patients, who received TL as primary treatment, 78% received adjuvant RT. There was no significant difference in use of adjuvant RT between the two decades and no significant difference in 5-year OS after TL compared to TL + RT (36 and 34%, respectively; *p* = 0.76). However, the TL + RT group included more T3–T4 (83%) than T1–T2 tumors (27%) as compared to the TL alone group (T3T4 64%, T1T2 36%). In the further analyses, no distinction was made between these two TL subgroups.

### Overall survival

The 5-year overall survival for the entire group (*n* = 2999) was 29%, including patients, who were not treated. When analyzed separately for patients who received RT, CRT or TL (± RT) (*n* = 2630), this was 31%. The 5-year OS for CRT (34%) and TL (34%) was significantly higher than RT (28%, *p* < 0.001), and there was no statistical significant difference in 5-year OS between CRT and TL for the total group. For the treated patients, 5-year OS increased significantly from 28% in the first decade to 34% in the second decade (*p* = 0.002). The small number of patients, who did not receive oncological treatment, had a 5-year OS rate of 3%. The patients who received only CT had a 5-year OS of 15%, and for the patients treated with surgery other than TL this was 46%. eTable 1 shows the 5-year OS rate stratified per treatment and TNM classification.

### Trends in overall survival for T1–T2 hypopharynx cancer

When stratified by treatment modality and TNM classification, the 5-year OS of T1–T2 patients treated with TL, CRT and RT was 40, 44, and 39%, respectively (*p* = 0.268). There was no significant difference in 5-year OS after TL or CRT between the two decades (TL 41% for 1991–2000 and 37% for 2001–2010, *p* = 0.92; CRT 40 and 46%, *p* = 0.353). For patients receiving primary RT, the 5-year OS increased significantly from 34% in the first to 42% in the second decade (*p* = 0.007).

### Trends in survival for T3 hypopharynx cancer

**Fig. 3:**
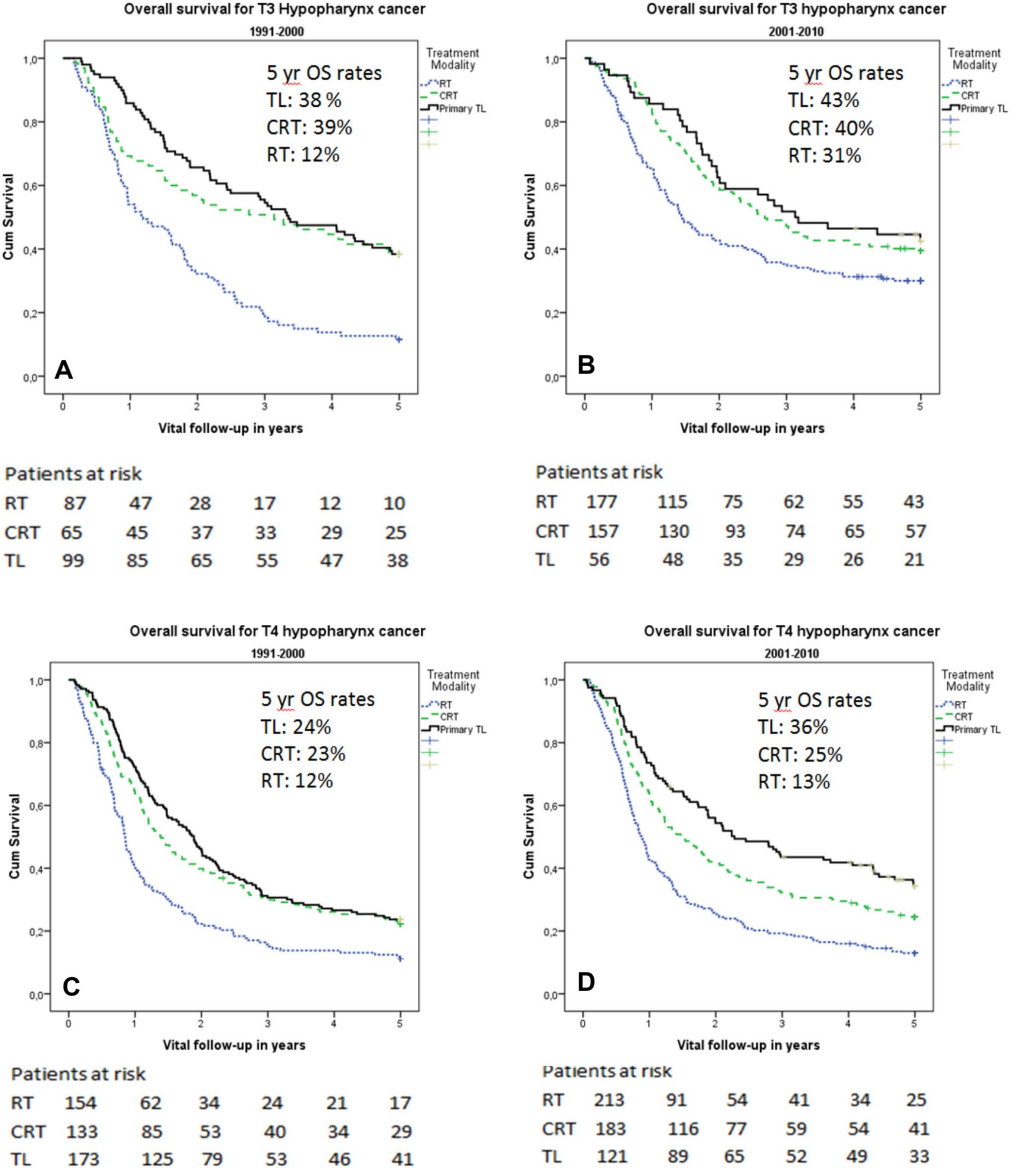
**a–d**. Kaplan–Meier OS curves. Kaplan–Meier OS rates for T3 hypopharynx cancer diagnosed in the first decade (**a**) or second decade (**b**) and T4 hypopharynx cancer diagnosed in the first (**a**) or second (**b**) decade

For patients with T3 hypopharynx cancer, the 5-year OS for TL and CRT did not differ significantly (40% and 39%, respectively; *p* = 0.475). The 5-year OS survival following radiotherapy (24%) was significantly poorer than for TL and CRT (*p* < 0.001). When comparing the two decades, 5-year OS following TL and CRT increased, but was not significantly better in the second decade (TL 38% for 1991–2000 and 43% for 2001–2010; *p* = 0.736, CRT 39% and 40%, respectively,  *p* =  0.664). The OS following RT increased significantly from 12% in the first decade to 31% in the second (*p* = 0.008). Kaplan–Meier curves of 5-year OS for T3 per decade are shown in Fig. 3a, b.

### Trends in survival for T4 hypopharynx cancer

For patients with T4 hypopharynx cancer, the 5-year OS was significantly better following a TL (29%) when compared to CRT (24%) (*p* = 0.039). Radiotherapy had the lowest 5-year OS of 13%. When comparing the two decades, there was a trend towards an improved 5-year OS after TL, which increased from 24% (1991–2000) to 36% (2001–2010) (*p* = 0.050). For RT and CRT, OS was not significantly different between the two decades (RT: 12 and 13%, *p* = 0.491; CRT: 23 and 25%, *p* = 0.682). Kaplan–Meier curves of 5-year OS for T4 per decade are shown in Fig. 3c, d.

### Salvage laryngectomy

During the study period, 706 TLs were performed: 567 primary TLs, 119 salvage TLs, 19 TLs for a dysfunctional larynx. One patient developed a second primary hypopharynx cancer 9 years after RT for a T2N0 posterior pharyngeal wall tumor that occurred in the same subsite, for which she underwent TL.

For the calculation of cumulative incidence of salvage laryngectomy, time in days was used starting from date of incidence until salvage/functional TL, death or date of last FU (cut off at 5-year), and patient status at 5-year (alive, dead, lost to follow-up). The cumulative incidence of salvage/functional TL at 5-year is 7% for RT and 4% for CRT (*p* = 0.02). The cumulative incidence of death at 5-year is 68% for RT and 64% for CRT (*p* = 0.006), see eFigure 1 for the cumulative incidence plot of TL and death, stratified by treatment (RT or CRT).

Of the 2063 patients initially treated with RT or CRT, 119 TLs were performed within 5 years after diagnosis; 87 for patients treated with RT and 32 for patients treated with CRT. The median time in days until salvage TL was 423, 400 days after RT and 469 days after CRT (*p* = 0.78).

### Multivariable analysis

We conducted a multivariable analysis to estimate the hazard ratio (HR) for death controlling for age, sex, TNM classification, treatment and subsite (Table 2). Receiving RT as primary treatment was associated with a significant higher risk of death when compared to TL (HR 1.59, 95% CI 1.40–1.81, *p* < 0.0001), which was not confirmed for CRT compared to TL in the total group (HR 1.07, 95% CI 0.93–1.23). We subsequently analyzed whether the time period (1991–2000 versus 2001–2010) had an impact on 5-year OS. Corrected for age, sex, TNM-stage, treatment (TL, RT or CRT) and subsite, the HR for death in the second decade was significantly lower than in the first decade both for T1–T2 tumors (HR 0.82, 95% CI 0.71–0.96, *p* = 0.01), and for T3–T4 tumors (HR 0.84, 95% CI 0.76–0.94, *p* = 0.002). In the previously described Kaplan–Meier analyses, we saw an increased 5-year OS rate for T4 tumors, increasing from 24% in the first to 36% in the second decade. When analyzing only T4 tumors treated in the second decade by a multivariate analysis, we found a significantly higher HR for death for CRT as compared to TL (HR 1.41, 95% CI 1.06–1.87, *p* = 0.02) when corrected for age, sex, TNM classification and subsite.

**Table 2:** Multivariable analysis for overall survival using Cox regression analysis including all patients treated with RT, CRT or TL

	T1–T4			T1–T2			T3–T4		
	HR	95% CI	*p* value	HR	95% CI	*p* value	HR	95% CI	*p* value
Age									
< 50	REF			REF			REF		
50–59	0.95	0.81–1.11	0.50	0.94	0.71–1.24	0.67	0.95	0.78–1.14	0.57
60–69	0.97	0.83–1.13	0.67	1.15	0.88–1.51	0.31	0.87	0.73–1.06	0.17
> 70	1.34	1.14–1.57	< **0.0001**	1.71	1.29–2.26	< **0.0001**	1.18	0.97–1.44	0.11
Sex									
Female	REF			REF			REF		
Male	1.17	1.04–1.32	**0.01**	1.23	1.00–1.52	0.05	1.15	0.96–1.34	0.06
TNM classification									
T1N0	REF			REF			–	–	–
T1N+	1.36	0.98–1.88	0.07	1.50	1.07–2.09	**0.02**	–	–	–
T2N0	1.01	0.75–1.38	0.93	1.03	0.76–1.41	0.84	–	–	–
T2N+	1.67	1.25–2.22	< **0.001**	1.75	1.30–2.34	< **0.001**	–	–	–
T3N0	1.43	1.02–1.99	**0.04**	–	–	–	REF		
T3N+	2.14	1.61–2.86	< **0.0001**	–	–	–	1.47	1.16–1.85	**0.001**
T4N0	2.12	1.57–2.87	< **0.0001**	–	–	–	1.49	1.16–1.91	**0.002**
T4N+	3.36	2.54–4.44	< **0.0001**	–	–	–	2.28	1.83–2.85	< **0.0001**
Treatment									
TL	REF			REF			REF		
RT	1.59	1.40–1.81	< **0.0001**	1.05	0.82–1.36	0.69	1.80	1.55–2.08	< **0.0001**
CRT	1.07	0.93–1.23	0.34	0.85	0.63–1.15	0.28	1.10	0.94–1.28	0.22
Subsite									
Pyriform sinus	REF			REF			REF		
Post-cricoid region	1.29	1. 06–1.58	**0.01**	1.58	1.12–2.22	**0.008**	1.18	0.92–1.51	0.20
Aryepiglottic fold	0.95	0.72–1.26	0.72	0.83	0.58–1.21	0.33	1.19	0.77–1.81	0.43
Posterior wall	1.31	1.10–1.56	**0.002**	1.46	1.10–1.92	**0.008**	1.23	0.98–1.54	0.08
Hypopharynx OL	1.43	1.13–1.79	**0.003**	0.83	0.47–1.48	0.53	1.56	1.21–2.01	**0.001**
Hypopharynx NOS	1.20	1.01–1.43	**0.04**	0.76	0.52–1.12	0.16	1.36	1.12–1.66	**0.002**

The given hazard ratios are hazard ratios for death

*HR* hazard ratio, *TL* total laryngectomy, *RT* radiotherapy, *CRT* chemoradiotherapy, *OL* overlapping, *NOS* not otherwise specified

## Discussion

This population-based study is one of the largest surveys on hypopharynx cancer published in the literature. With it, we were able to answer the three main research questions posed in the introduction. First, we found that after an initial increase in ESR incidence from 1989 until 1997, there was a non-significant decline from 1997 to 2013. Second, we established that there was a significant decline in the use of TL for both T1–T2 and T3–T4 tumors over the two decades. Last, we found that the 5-year OS for all patients treated with RT, CRT and TL significantly improved in the second decade when compared to the first decade (28–34%, respectively). Moreover, we observed that T4 patients had the highest 5-year OS rate when treated with TL, followed by CRT and RT (29 versus 24 and 13%).

The trend towards a declining incidence in hypopharynx cancer observed in our cohort since 1997 is in line with international trends [20, 21], as are the changing treatment trends [9]. Lefebvre et al. were one of the first to evaluate organ preservation therapies for hypopharynx cancer in an RCT. The authors found no significant difference in OS, and concluded that organ preservation is the preferred treatment when the tumor is chemo-sensitive [22]. Although since then CRT has been routinely used in clinical practice, controversy remained. Meta-analyses specifically comparing TL to CRT for hypopharynx cancer are not available. In 2000, Pignon et al. described a meta-analysis on larynx preservation based on 3 RCTs of which only one RCT included hypopharynx cancer patients. They demonstrated a reduced survival in the CRT arm of 6% at 5 years when compared to TL [23]. In 2011, a meta-analysis analyzing the addition of chemotherapy to locoregional treatment was published in which hypopharynx cancer was analyzed separately. Loco-regional treatment could be: standard/hyperfractionated RT, surgery (with or without RT) or ‘other’. For patients with hypopharynx cancer, an overall survival benefit at 5 years of 4% was observed when chemotherapy was added to any loco-regional treatment [24]. However, again there was no direct comparison between CRT and TL in this meta-analysis.

Despite the improved prognosis in the last decade, OS for patients with hypopharynx cancer remains poor [4, 21, 25]. Up to 95% of all recurrences occur in the first 36 months and over half of the first recurrences are distant metastases [26]. Our National Cancer Registry database did not collect data regarding (logo-regional) recurrences and the development of distant metastasis, precluding us from drawing conclusion on these issues. In other cohorts, the incidence of distant metastases constitutes a large problem among hypopharynx cancer patients affecting between 9–40% of patients during follow-up [4, 26–29]. Another issue among hypopharynx cancer patients is the low number of salvage TLs performed after failed RT or CRT. The low cumulative incidence of 4–7% of salvage/functional TL at 5-year mainly reflects the incurability of most recurrences, reflected by the low 5-year OS (RT 28%, CRT 34%), and supported by low OS rates after salvage TL for hypopharynx cancer [30]. Furthermore, as patients diagnosed and treated with (C)RT before 1990 were not included in our database, the patients at risk for salvage TL or TL for a dysfunctional larynx in the first few years are not representative of the actual number of patients at risk in those years.

In concordance with other studies concerning hypopharynx cancer, we demonstrated an increased OS for female patients [21]. Possibly a combination of biological and medical behavior plays a role; however, with the results from our study, this remains speculation. In 2010, a matched pair analysis of patients treated for head and neck cancer in the Memorial Sloan Kettering center was performed to evaluate gender survival disparities. After matching 286 men and 286 women on 6 known prognostic variables, the authors concluded that there is no difference between men and women in recurrence-free, disease-specific or overall survival [31]. Possibly, female patients in our cohort indeed had favorable prognostic variables that contributed to their superior OS.

In our cohort, we found no significant difference in OS between TL and CRT for T3 tumors, whereas both treatments had higher OS rates when compared to RT alone. For T4 tumors, we did find a significant difference, where TL was associated with the highest 5-year OS rates when compared to CRT and RT. Several other authors have reported higher 5-year OS rates following TL as compared to RT [7, 21, 25]. Furthermore, a large epidemiological study in the USA reported worse outcomes for CRT compared to TL [4]. Due to the observational nature of all retrospective studies, the results, in part, can be confounded by indication. Yet, given the lack of robust evidence for equivalence of organ preservation compared to TL in patients with T4 tumors, and the observed better survival after TL in epidemiological cohort studies, the assumed equivalence of CRT and TL for T4 tumors is questionable. In our opinion, TL should not be restricted to those patients who carry a high risk on a dysfunctional larynx after (C)RT, but all T4 patients should be counseled on the potentially better chance for survival using TL, as compared to (C)RT.

### Limitations

The results of our study have to be interpreted with caution. Despite the fact that we used data collected by trained cancer registry administrators, the accuracy of the NCR data as such cannot be tested. However, by combining the NCR database with the national Pathology database, we were able to verify the histopathology of the tumors and the treatment modalities. However, details regarding treatment, patient- and tumor characteristics are lacking in this national database. Some patients, especially in the T4 group, might have received RT with palliative intent. Despite these considerations, the conclusions on the trends in incidence, treatment and OS of hypopharynx cancer from this large population-based database seem valid.

## Conclusion

This population-based study demonstrates a shift in treatment preference towards organ preservation therapies in the Netherlands, with a significant decline in TL and a significant increase in RT and CRT since 2001. At the same time, the 5-year OS of patients treated with RT, CRT or TL increased significantly. Based on our results, the assumed equivalence of CRT and TL for T4 disease may require reconsideration.

## Electronic supplementary material

Below is the link to the electronic supplementary material.

Supplementary material 1 (DOCX 234 KB)
