# Supplementary material for: Trends in treatment, incidence and survival of hypopharynx cancer: a 20-year population-based study in the Netherlands
Source: Eur Arch Otorhinolaryngol. 2017 Oct 28;275(1):181–9. doi: 10.1007/s00405-017-4766-6 (PMC5754418; doi:10.1007/s00405-017-4766-6)
Supplement: Supplementary file 1 — Supplementary material 1 (DOCX 234 KB) [file 405_2017_4766_MOESM1_ESM.docx]

**Supplementary material**

**eFigure 1.** Cumulative incidence of incidence of salvage/functional TL or death.


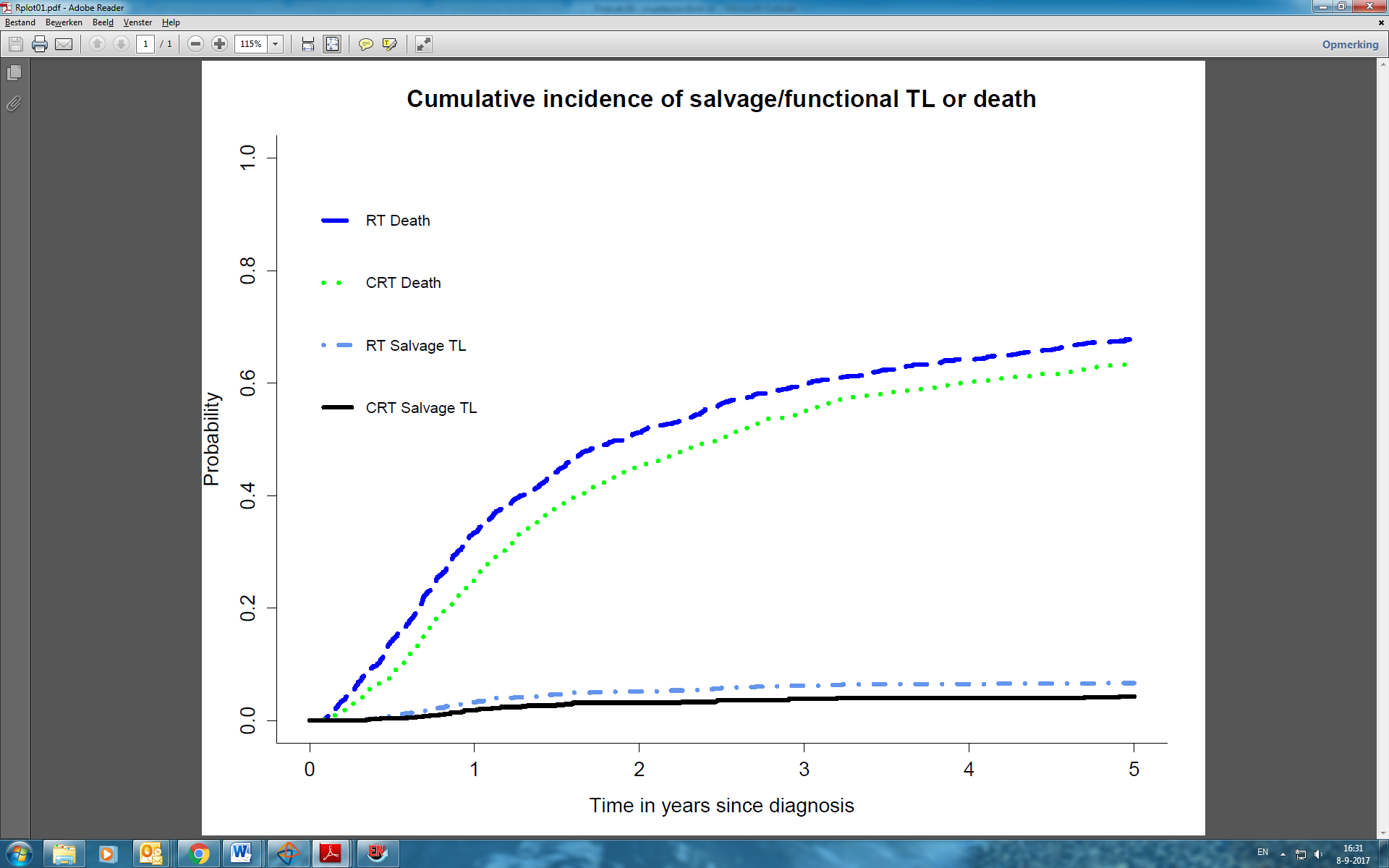


The X-asis depicts the time in years since diagnosis, the Y-axis depicts the cumulative probability on a salvage/functional TL or death.

**Supplementary table 1.** 5-Year OS rate per treatment modality and TNM-classification

| TNM classification | Treatment Modality | 5-year OS  1991-2010 | N | P value |
| --- | --- | --- | --- | --- |
| T1N0 | RT | 46% | 85 |  |
|  | CRT | 50% | 6 |  |
|  | TL | 56% | 18 | 0.736 |
| T1N+ | RT | 39% | 128 |  |
|  | CRT | 41% | 27 |  |
|  | TL | 38% | 8 | 0.959 |
| T2N0 | RT | 44% | 194 |  |
|  | CRT | 69% | 36 |  |
|  | TL | 50% | 44 | **0.025** |
| T2N+ | RT | 32% | 273 |  |
|  | CRT | 37% | 145 |  |
|  | TL | 25% | 48 | 0.091 |
| T3N0 | RT | 36% | 75 |  |
|  | CRT | 59% | 39 |  |
|  | TL | 42% | 48 | **0.024** |
| T3N+ | RT | 20% | 189 |  |
|  | CRT | 35% | 183 |  |
|  | TL | 39% | 107 | **<0.001** |
| T4N0 | RT | 20% | 111 |  |
|  | CRT | 38% | 69 |  |
|  | TL | 38% | 101 | **<0.001** |
| T4N+ | RT | 9% | 256 |  |
|  | CRT | 20% | 247 |  |
|  | TL | 24% | 193 | **<0.001** |

N= number of patients, P value was calculated between the three treatment options per TNM-classification (Log Rank)
